# Comparison of ultrasound phacoemulsification and FemtoMatrix^®^ PhotoEmulsification^®^ cataract surgery

**DOI:** 10.3389/fmed.2023.1157486

**Published:** 2023-04-17

**Authors:** Amélie de Saint Jean, Damien Dufournel, Pavel Stodulka, Fabrice Romano, Aurélien Bernard

**Affiliations:** ^1^KERANOVA, Saint Etienne, France; ^2^Gemini Eye Clinic, Zlín, Czechia; ^3^Third Faculty of Medicine, Charles University, Prague, Czechia

**Keywords:** cataract surgery, femtosecond laser, FemtoMatrix^®^, PhotoEmulsification^®^, safety, efficacy

## Abstract

**Objective:**

To introduce a novel technology currently under final development before regulatory approvals for the furtherment of cataract surgery, using the FemtoMatrix^®^ laser system, and to demonstrate its safety and efficacy as compared to standard ultrasound phacoemulsification.

**Methods:**

Thirty-three patients with bilateral cataracts were operated on with one eye undergoing PhotoEmulsification^®^ treatment on the FemtoMatrix^®^ device and the contralateral eye receiving the control procedure, i.e., standard ultrasound phacoemulsification treatment. The number of “zero-phaco” procedures (denoting that I/A alone was sufficient to aspirate the lens fragments and that no ultrasound energy was needed) was recorded and Effective Phaco Time (EPT) values were compared. The patient follow-up was 3 months.

**Results:**

Thirty-three eyes from a population with a mean cataract grade of 2.6 were treated on the FemtoMatrix^®^, of which 29 were “zero-phaco” (88%). All patients were operated on by a single surgeon who was a relative novice to the technology (63 patients treated prior to this study). Conversely, of the 33 fellow eyes who underwent standard ultrasound phacoemulsification, none were zero-phaco (0%) - all required varying degrees of ultrasound energy to make lens aspiration possible. The mean EPT was significantly lower in the PhotoEmulsification^®^ laser group (0.2 ± 0.8 s) than in the phaco group (1.3 ± 1.2 s) (*p* < 0.0001). The safety profiles of the two procedures were comparable, with no device-related adverse events noted.

**Conclusion:**

FemtoMatrix^®^ is a promising femtosecond laser platform that, when compared to phacoemulsification, significantly decreases or eliminates EPT altogether. The system is used to perform PhotoEmulsification^®^, making zero-phaco cataract procedures feasible including in high-grade cataracts (>3). It enables personalized treatment by automatically measuring and adapting the laser energy required to obtain the most efficient cutting of the crystalline lens. This new technology appears to be safe and effective in cataract surgery.

## 1. Introduction

According to World Health Organization, cataracts constitute the leading cause of reversible blindness globally and also represent the second most common cause of moderate and severe vision impairment (1). Currently, cataracts can only be addressed surgically and the procedure is one of the most frequently practiced, with rates on the rise worldwide (over 28 million surgeries globally in 2019 (2)). Phacoemulsification Cataract Surgery, developed around half a century ago, remains to this day the gold standard (accounting for nearly 70% of global cataract procedures) (3, 4). However, with the development of Femtosecond Laser-Assisted Cataract Surgery (FLACS) over the course of the last decade, one of the primary objectives was to reduce to the greatest extent possible recourse to ultrasound with the ultimate goal of achieving what Abell et al. coined the “zero-phaco” procedure (5). Despite considerable efforts made by the industry, this goal remains, as of yet, unfulfilled.

**Figure 1:**
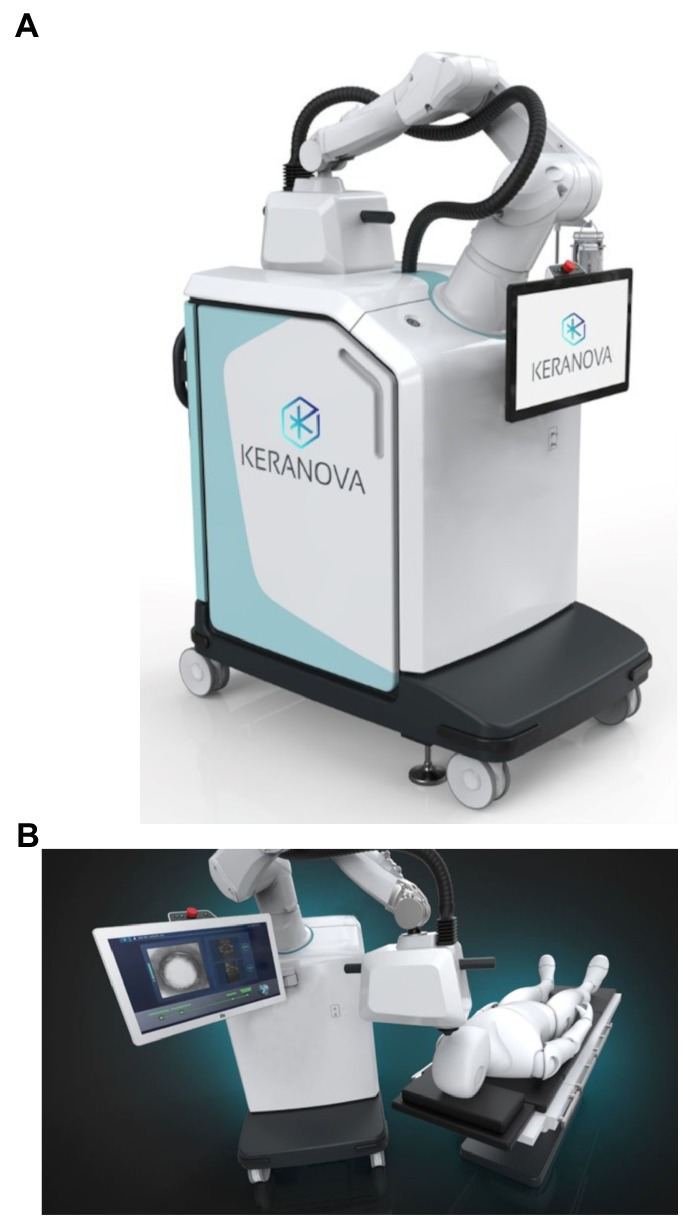
FemtoMatrix^®^, a new robotic surgery system that uses femtosecond laser technology to treat cataracts. **(A)** Side view of the device, **(B)** view of the functioning device.

**Figure 2:**
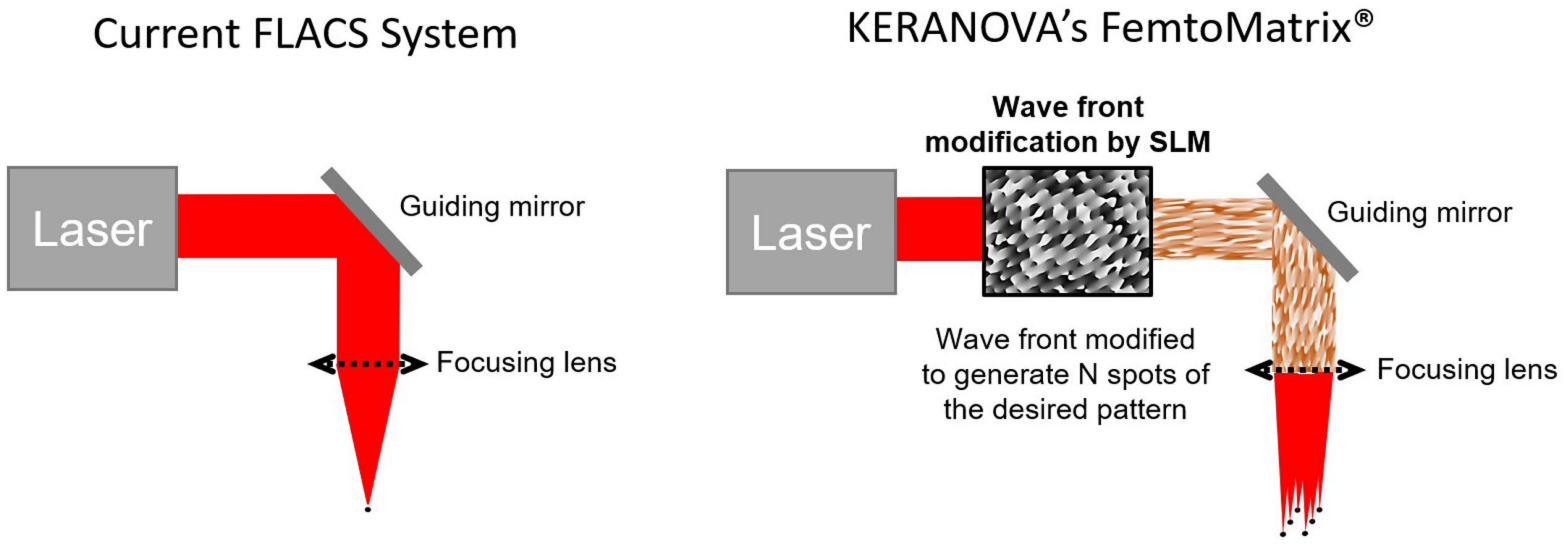
Comparison of laser spot generation for current FLACS versus FemtoMatrix^®^.

**Figure 3:**
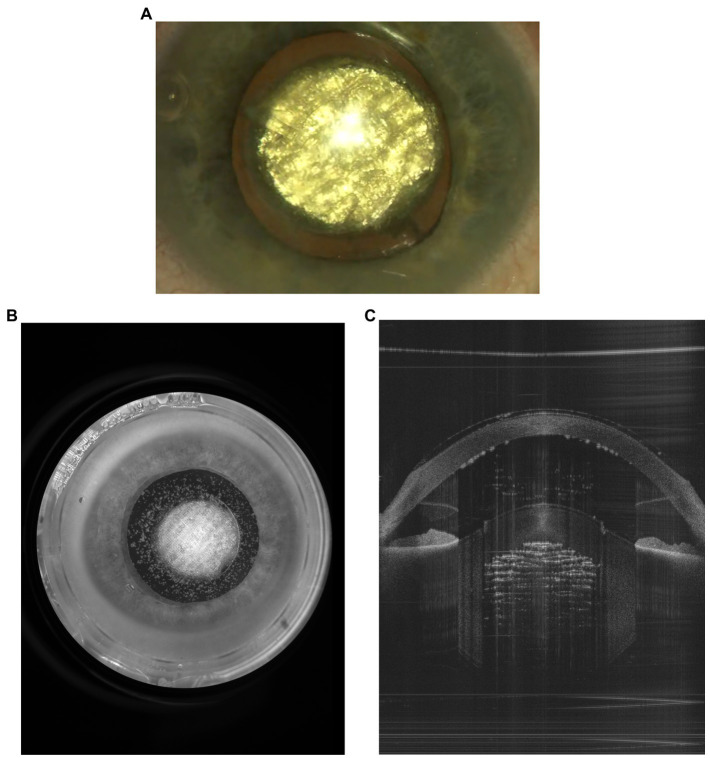
PhotoEmulsification^®^ of a cataracted lens producing 200-μm cubes sized. **(A)** View from the surgical microscope, **(B)** capture from the FemtoMatrix^®^ system (Frontview camera), **(C)** capture from the FemtoMatrix^®^ system (OCT).

In the present article, a new generation of femtosecond lasers currently in development is presented: the FemtoMatrix^®^ (see Figure 1). This mobile system can be installed and used in the operating room. It possesses photonic capability to drastically accelerate laser speed utilizing a Spatial Light Modulator (SLM) designed to modify, in real-time, the wavefront of the laser beam. During laser treatment, this enables - for the first time ever - reshaping the laser beam in the focal plane into a multispot matrix or variable geometry spots (Figure 2) thereby allowing the FemtoMatrix^®^ to perform cuts at much higher speeds than the current standard of FLACS systems. The resulting effect is PhotoEmulsification^®^, incising the crystalline lens nucleus into a grid-like pattern of tiny cubes (see Figure 3). The surgeon may then remove the particles through simple irrigation/aspiration in an overwhelming majority of cases.

The FemtoMatrix^®^ also incorporates an intelligent and autonomous OCT image computing-based system that calculates the ideal energy level necessary for safe and effective emulsification thereby reducing the amount of gas produced and allowing the surgeon to perform personalized treatment including in tougher cataract grades (>3). The customized energy level is determined with the help of intelligent software OCT observation of the actual effect of increasing energy levels in multiple test areas performed by the laser within the lens in a matter of seconds, before the PhotoEmulsification^®^ treatment starts, unlike FLACS systems which calculate energy levels based on nomograms based on the optical density of the lens which is only one of many parameters than can affect the efficiency. We report, herein, the results of a clinical trial that pits the performance and safety of the FemtoMatrix^®^ system against ultrasound phacoemulsification with a focus on Effective Phaco Time (EPT), as a comparator.

## 2. Methods

### 2.1. Patients and methods

Thirty-three patients with age-related bilateral cataract were operated on by a single surgeon (PS) at one site (Gemini Eye Clinic, Zlín, Czech Republic), from May 9 to 12, 2022. Treatment was randomly attributed with one eye treated with the FemtoMatrix^®^ and the other receiving the control procedure, i.e., standard ultrasound phacoemulsification treatment. This clinical investigation was conducted in accordance with the ethical principles per the Declaration of Helsinki; Ethical Principles for Medical Research Involving Human Subjects, and the ISO 14155:2011 standard; Clinical Investigations for Medical Devices and began after receiving approval from the Ethical Committee and the Health Authorities of the Czech Republic (EUDRA CT Number: 2021-006651-34).

Inclusion criteria included: patients aged at least 40 presenting bilateral cataracts impairing Corrected Distance Visual Acuity (CDVA ≤0.8). Exclusion criteria included previous ophthalmic surgery (including refractive surgery), ophthalmic pathologies such as glaucoma, corneal scars, pterygium, irregular astigmatism, corneal degeneration, or dystrophy, retinal pathology, recent history of uveitis or optic neuropathy and uncontrolled ocular or systemic disease (e.g., diabetes mellitus, active cancer treatment, mental illness, etc.). Furthermore, patients with poorly dilating pupils (mydriatic pupil size ≤5 mm) and/or a baseline Endothelial Cell Density (ECD) ≤1,500 cells/mm^2^, were not considered for inclusion.

### 2.2. Pre-operative assessment

All patients underwent a baseline preoperative assessment including routine examinations such as visual acuity, slit lamp examination, IOP measurement, keratometry, pachymetry, biometry and Intraocular Lens (IOL) power calculation, macular scan of optical coherence tomography and specular microscopy.

Additionally, the following data was collected: age, gender, visual acuity, intraocular pressure (IOP), axial length, endothelial cell count, corneal curvature, corneal thickness, foveal thickness, cataract type [lens opacities classification system III, LOCS III grades (6)], and general health status.

### 2.3. Surgical procedure

All surgeries were conducted under topical anesthesia in an ambulatory surgery setting, per standard site practice. Both eyes were operated on the same day always starting by the right eye. Treatment was randomly assigned with one eye undergoing PhotoEmulsification^®^ on the FemtoMatrix^®^ device while the contralateral eye received the control procedure, i.e., ultrasound phacoemulsification using the Stellaris phaco machine (Bausch + Lomb, Rochester, NY, United States).

Regarding the FemtoMatrix^®^ (KERANOVA, Saint-Etienne, France), the main steps of the PhotoEmulsification^®^ procedure performed are, sequentially, as follows:

- Docking, i.e., placement on the eye of the two-piece docking kit which includes a patient interface incorporating a suction chamber and a curved lens made of hydrogel
- Image computing *via* the front-view camera image and two OCT images to enable automatic recognition of key ocular structures to program the cutting patterns
- Automatic laser energy setting by means of the Threshold Scanning System (TSS) tool, an intelligent, autonomous OCT image-based system which calculates the ideal energy level necessary for the PhotoEmulsification^®^ with an optimized cutting efficiency/gas produced ratio
- Capsulotomy (diameter adjustable from 4,000 μm to 7,000 μm, preset at 4,800 μm upon surgeon request and adjusted from 4,000 to 4,800 depending on the pupil size) performed top-down using a monospot at 3 μJ per spot
- PhotoEmulsification^®^ (crystalline lens nucleus diced into 200 μm cubes using a shaped laser beam and energy which can be adjusted from 1 μJ to 6.6 μJ per spot)
- Dedocking of the patient eye.

All automatic steps are manually changeable should the User deem it necessary.

On the fellow eye, a sham docking was performed to mimic a FemtoMatrix^®^ procedure. Patients were thus unaware which treatment they were receiving.

After the FemtoMatrix^®^ procedure and sham docking, the surgeon proceeded with the rest of the surgery with the patient on the same surgical bed. This is a major advantage compared to current FLACS machines as patient workflow is not disrupted. Moving the patient is unnecessary between the two phases surgery, rendering the procedure faster and safer for the patient. This is due to the use of a robot which assists the surgeon to avoid numerous manipulations, and automatically shifts the laser head to the patient. Corneal incisions were performed manually. The anterior chamber was filled with a viscoelastic agent. In the standard ultrasound phacoemulsification cases, a capsulorhexis was performed using forceps whereas in the PhotoEmulsification^®^ group, the surgeon removed the anterior capsule which had been precut by the FemtoMatrix^®^. Hydrodissection was performed solely in the standard ultrasound phacoemulsification group. Next, in both groups the crystalline lens material was extracted using the same BAUSCH + LOMB phaco handpiece (tip reference: BL3318, 30° MICS™, Straight needle, RIS 1.80–2.00 mm, OD: 0.74–0.95 mm, ID: 0.50–0.79 mm) with or without ultrasound (Stellaris phacoemulsification device, BAUSCH + LOMB, Rochester, NY, United States). IOL insertion was conducted similarly in both eyes of each patient using the corresponding injector for each IOL.

### 2.4. Follow-up

The patients were followed up for 3 months. Visual acuity and IOP measurement, slit lamp examination, pachymetry, an OCT scan of the macula, and specular microscopy were all conducted postoperatively. The following data was collected: visual acuity, IOP, endothelial cell count, corneal thickness, and any complications.

### 2.5. Outcomes

Performance was evaluated based on the comparison between the two groups of the following criteria:

- The percentage of eyes receiving a zero-phaco procedure
- Mean EPT (intraoperative measurements)
- CDVA at 3 months.

Customization of the laser treatment thanks to the TSS was evaluated by comparing the nucleus grade of the cataract and the mean energy per spot used for the PhotoEmulsification^®^.

Safety was evaluated based on:

- The occurrence of adverse events ascribable to the device
- Postoperative outcomes including Endothelial Cell Count (ECC) and Central Corneal Thickness (CCT) at 3 months.

### 2.6. Statistical analysis

GraphPad Prism 9.4.1 for Windows (San Diego, CA, United States) was used to perform the statistical analysis. We compared sample means on parametric and non-parametric data sets using *T*-tests or Wilcox on matched pairs tests. Fisher’s exact test and Mann–Whitney test were used to compare distributions (for example, the number of zero-phaco procedures according to treatment and/or cataract grade). Statistical significance was defined as a *p*-value under 0.05.

## 3. Results

The thirty-three patients enrolled were all bilaterally operated with one eye treated by the FemtoMatrix® and the other by standard ultrasound phacoemulsification, per the protocol. The population was composed of 16 females and 17 males, ranging in age from 46 to 86 years (61 ± 9.4 on average). Baseline characteristics of patients are provided in Table 1. There was no statistically significant difference for the mean cataract grade (based on the Nc of the LOCS III, *p* = 0.4660) or in the preoperative routine eye examination results between the two groups.

**Table 1:** Baseline characteristics of the patients.

Parameter	FemtoMatrix^®^ (*n* = 33)	Standard ultrasound phacoemulsification (*n* = 33)	*p*-value in italics when statistically significant
LOCS classification mean value (Nc)	2.6 (1.1; 0.6–5.0)	2.6 (1.2; 0.6–5.0)	0.4660^b^
Mean IOP (mmHg)	14.5 (3.3; 10.0–21.0)	14.6 (3.5; 9.0–21.5)	0.7051^a^
ECC (cells/mm^2^)	2,604 (297; 1,686–3,037)	2,627 (292; 1741–3,050)	0.1641^b^
CCT (μm)	558 (26; 506–605)	556 (20; 498–604)	0.4833^a^
Macular OCT foveal thickness (μm)	231 (20; 186–274)	232 (21; 198–271)	0.3842^a^
CDVA	0.65 (0.20; 0.16–0.80)	0.67 (0.21; 0.06–0.80)	0.3959^b^

Data are number of patients (%), mean (SD), or mean (SD; minimum – maximum). LOCS, lens opacities classification system; Nc, nuclear color, IOP, intra ocular pressure; ECC, endothelial cell count; CCT, central corneal thickness; OCT, optical coherence tomography; CDVA, corrected distance visual acuity. ^a^Paired *t*-test; ^b^Wilcoxon matched pairs.

The docking was easily performed as it was achieved on its first go in 97% of instances (32 of 33 patients) and on its second try when redocking was necessary.

Capsulotomies were all complete and considered as “very easy” to remove by the surgeon. No tear was reported neither in the FemtoMatrix^®^ group nor in the phaco one.

As set forth in Table 2, of the 33 eyes in the FemtoMatrix^®^ group, 29 were zero-phaco (88%) while there were none in the standard ultrasound phacoemulsification group (0%). Moreover, the mean EPT value was significantly lower (*p* < 0.0001) in the FemtoMatrix^®^ group (0.2 s ± 0.8) compared to standard ultrasound phacoemulsification (1.3 s ± 1.2). Postoperatively, improvement in CDVA was observed in both groups with a mean 0.98 ± 0.06 in the FemtoMatrix^®^ group and a mean 0.99 ± 0.08 for eyes undergoing phaco (no statistically significant difference between the two populations, see Table 3).

**Table 2:** Intraoperative parameters in the two groups of the analyzed sample.

Parameter	FemtoMatrix^®^ (*n* = 33)	Standard ultrasound phacoemulsification (*n* = 33)	*p*-value in italics when statistically significant
Zero phaco-procedure	29 (88%)	0 (0%)	<0.0001^a^
Effective phaco time (seconds)	0.2 (0.8; 0.0–4.5)	1.3 (1.2; 0.3–6.0)	<0.0001^b^

Data are number of patients (%), mean (SD), or mean (SD; minimum – maximum). ^a^Fisher’s exact test; ^b^Wilcoxon matched pairs.

**Table 3:** Postoperative characteristics of the patients.

Parameter	FemtoMatrix^®^ (*n* = 33)	Standard ultrasound phacoemulsification (*n* = 33)	*p*-value in italics when statistically significant
Mean IOP (mmHg)	12.3 (3.5; 6.0–20.0)	12.5 (3.2; 5.0–18.0)	0.5922^a^
ECC (cells/mm^2^)	2,348 (428; 1,024–2,860)	2,409 (479; 1,083–3,019)	0.1754^b^
CCT (μm)	560 (26; 510–606)	558 (28; 505–604)	0.5074^a^
Macular OCT foveal thickness (μm)	236 (24; 154–277)	236 (23; 176–276)	0.9567^a^
CDVA	0.98 (0.06; 0.70–1.00)	0.99 (0.08; 0.80–1.25)	0.8438^b^

Data are mean (SD), or mean (SD; minimum – maximum). IOP, intra ocular pressure; ECC, endothelial cell count; CCT, central corneal thickness; OCT, optical coherence tomography; CDVA, best corrected distance visual acuity. ^a^Paired *t*-test; ^b^Wilcoxon matched pairs.

**Figure 4:**
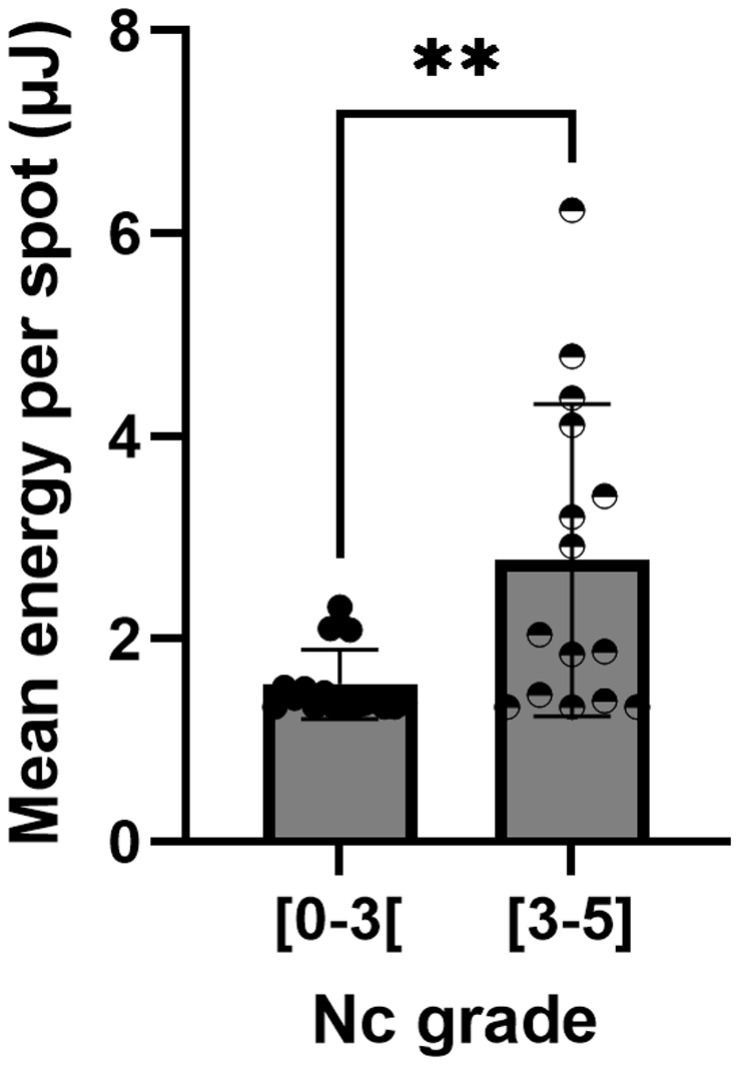
Mean energy sent per spot according to the nuclear color cataract grade.

**Figure 5:**
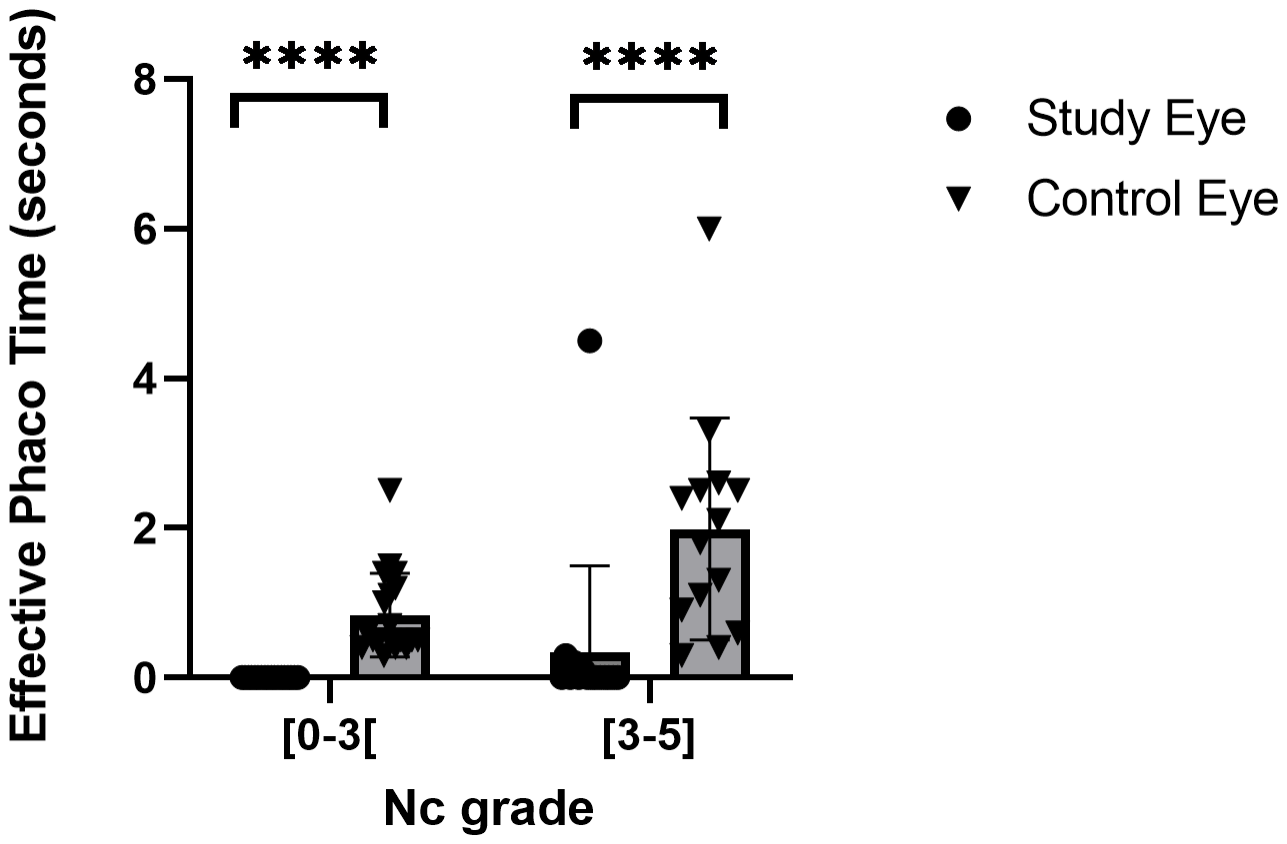
Effective phaco time according to the nuclear color cataract grade.

In terms of the customization of treatment through automatic adjustment of laser energy, Figure 4 shows that TSS measured significantly lower energy required to cut the lens for grades between 0 and 2.9 compared to cataract grades 3 and higher the mean energy per spot used in cases where the Nc grade was between 3 and 5 was 1.8 times higher than in eyes with a Nc grade between 0.6 and 2.9. It is noteworthy that the procedures performed with the FemtoMatrix^®^ on patients with a cataract grade under 3 were all zero-phaco procedures (*N* = 18), with a mean energy per spot of 1.6 μJ. Seventy-three per cent of the surgeries performed with the FemtoMatrix^®^ on patients with a cataract grade between 3 and 5 were zero-phaco procedures (*N* = 11/15) with a mean energy per spot of 2.8 μJ. Only one patient required phaco where EPT >0.3 s. Furthermore, when comparing the EPT values according to nuclear grade, as shown in Figure 5, modulation of the energy does not appear to compromise the efficiency of the treatment since, irrespective of the nuclear grade, EPT in the FemtoMatrix^®^ group was consistently much lower than that in the standard ultrasound phacoemulsification patient series. For instance, if the population with a grade <3 (average = 1.8, *N* = 18) is considered, and the EPT between FemtoMatrix^®^ eye and standard ultrasound phacoemulsification eye is compared, the reduction in EPT is 100%. Further, if the population with a grade ≥3 (average = 3.5, *N* = 15) is considered and again the EPT between FemtoMatrix^®^ eye and standard ultrasound phacoemulsification eye is compared, the reduction in EPT is 74%, and only 4 patients received ultrasound energy and in 3 of the four the EPT was less than 0.3 s, which means that they were used only with very short bursts to unclog the tip.

In respect to safety profiles, there were no adverse events considered to be associated with the FemtoMatrix^®^. There were no inadvertent dedockings. More significantly, there were no capsular ruptures. Lastly, no significant differences in terms of postoperative outcomes between the two groups were reported at 3 months, as shown in Table 3.

## 4. Discussion

PhotoEmulsification^®^ is a fundamentally new solution in cataract surgery. This article presents the results from the first clinical study that compares this new technology to the current gold standard, i.e., standard ultrasound phacoemulsification. The results demonstrates that the emerging photonic technology compares favorably to the established worldwide-used phacoemulsification method.

Capsulotomies were all complete and very easy to remove without capsular tear. It seems that the top-down approach avoids cutting the anterior cortex and creating adhesions between the cortex and the rim of the capsulotomy However, further dedicated studies are needed to support this observation.

Despite the low number of patients included (*N* = 33), this study clearly shows that zero-phaco procedure is an achievable goal including in higher grade cataracts (>3). Zero-phaco procedures were already described using LenSx^®^ (Alcon Laboratories, Fort Worth, TX, United States), the most widely used femtosecond laser platform today. However, the zero-phaco rates obtained were respectively: 80% of for Grade 0; 52% for Grade 1; 8% for Grade 2; zero phaco was not achievable in Grade 3 cataracts (7). In the present study, zero-phaco was systematically achieved for grades <3 where patients underwent treatment on the FemtoMatrix^®^. Where grade was ≥3, 11 of the 15 patients were zero-phaco procedures (73%), including one with Grade 4.5 and one Grade 5 cataract.

Treatment with the LenSx^®^ femtosecond laser platform was all performed using 5 μJ per spot (7) whereas the energy level with the FemtoMatrix^®^ varied from 1 to 6.6 μJ having been adapted for personalized treatment be means of the Threshold Scanning System (TSS). TSS is a patented feature that not only personalizes treatment for each patient but also allows to adjust the level of energy in the respective regions of the crystalline lens. This feature is based on intelligent real-time OCT image computing after several laser shots of increasing energy in 24 different locations throughout the lens, monitoring for the appearance of a gas bubble. This process takes a handful of seconds. It is thus a highly adaptable system that allow to obtain an efficient cutting without creating an excess of gas.

These promising results were obtained by an experienced cataract surgeon (PS) who, in addition to being a proficient phaco surgeon, is accustomed to using an existing FLACS system. Leading up to the present study, he had performed just 63 procedures with the FemtoMatrix^®^ or earlier prototypes, which is less than the usual 100 procedures considered to constitute a learning curve (8) and well below the 1,000 procedures described by Dick and Schultz to optimize the surgery parameters and the surgeon practice (9). On the other hand, we did note that PhotoEmulsification^®^ requires a slight adaptation of the surgeon’s technique. For instance, detaching and removing small lens fragments still attached to each other without ultrasound can be challenging if the surgeon follows their habitual gesture. Further studies are thus needed to conclude about the FemtoMatrix^®^ learning curve. However, we expect that the surgeon will attain a good level of proficiency after a modest learning curve. We anticipate that developing a custom-built instrument (tip) and describing some evolutions of the surgical technique would also considerably improve the efficiency of the procedure. And of course, like phacoemulsification at the very beginning, a new technique will benefit from the experience of the first users who will find probably many other ways to improve these new surgical gestures.

Out of the cohort of 33 patients, only 4 received ultrasound energy. In three of these the EPT observed was very low (0.1, 0.2 and 0.3 s). In substance, ultrasound bursts were used to unclog the flared phaco tip. All told, 97% of the overall population of patients received zero (88%) or an insignificant amount (9%) of ultrasound energy.

In spite of the difficulties to compare EPT between two platforms due to differences in algorithms or other confounding parameters (such as cataract grade or the level of surgeon experience), the FemtoMatrix^®^ clearly compares favorably to the other available FLACS devices such as the Catalys^®^ and the LenSx^®^. In a comparative study between these two machines, the reported mean U/S EPT values were, respectively, 6.8 and 8.9 s for mean grade 2 cataracts, compared to 0.2 s with the FemtoMatrix^®^. This translates into a factor of x34 and x44.5, respectively, versus the FemtoMatrix^®^ which was used on a population where the average cataract grade was 2.6 (10).

## 5. Conclusion

The FemtoMatrix^®^ is a new femtosecond laser platform under development by KERANOVA that appears to be safe and efficient. It represents promising technology to eliminate or radically decrease EPT during cataract surgery thereby making zero-phaco procedures possible, including in denser, higher-grade cataracts (>3).

## Data availability statement

The raw data supporting the conclusions of this article will be made available by the authors, without undue reservation.

## Ethics statement

The studies involving human participants were reviewed and approved by ETICKA KOMISE OCNI KLINIKY GEMINI. The patients/participants provided their written informed consent to participate in this study.

## Author contributions

DD designed the study. PS performed the surgeries. AS, DD, FR, and AB analyzed and interpreted the data. AS wrote the first draft of the paper. All authors contributed to the article and approved the submitted version.

## Conflict of interest

FR and AB are co-founders of KERANOVA. AS and DD are employees of KERANOVA. PS is the head-surgeon of Gemini Eye Clinics, member of KERANOVA Scientific board.

## Publisher’s note

All claims expressed in this article are solely those of the authors and do not necessarily represent those of their affiliated organizations, or those of the publisher, the editors and the reviewers. Any product that may be evaluated in this article, or claim that may be made by its manufacturer, is not guaranteed or endorsed by the publisher.

## References

[1] GBD 2019 Blindness and Vision Impairment Collaborators, Vision Loss Expert Group of the Global Burden of Disease Study. Causes of blindness and vision impairment in 2020 and trends over 30 years, and prevalence of avoidable blindness in relation to VISION 2020: the right to sight: an analysis for the global burden of disease study. Lancet Glob Health. (2021) 9:e144-60. doi: 10.1016/S2214-109X(20)30489-7 33275949 PMC7820391

[2] Eyewire. Cataract Surgery Products Continue to be the Main Single-use Surgical Device Sales Driver (Source: Market Scope). Eyewire+ Bryn Mawr Communication (2019). Available at: https://eyewire.news/news/cataract-surgery-products-continue-to-be-the-main-single-use-surgical-device-sales-driver (Accessed October 26, 2022)

[3] Grzybowski A Kanclerz P. Recent developments in cataract surgery In: Grzybowski A, editor. Current Concepts in Ophthalmology. Cham: Springer International Publishing (2020). 55–97.

[4] Eyewire. Market Scope: Phaco and FLACS Upgrades to Drive Expansion in Cataract Equipment Market. Eyewire+. Bryn Mawr Communications; (2021). Available at: https://eyewire.news/news/market-scope-phaco-and-flacs-upgrades-to-drive-expansion-in-cataract-equipment-market (Accessed October 26, 2022).

[5] Abell RG Kerr NM Vote BJ. Toward zero effective phacoemulsification time using femtosecond laser pretreatment. Ophthalmology. (2013) 120:942–8. doi: 10.1016/j.ophtha.2012.11.045, PMID: 23465860

[6] Chylack LT Jr Wolfe JK Singer DM Leske MC Bullimore MA Bailey IL. The lens opacities classification system III. The longitudinal study of cataract study group. Arch Ophthalmol. (1993) 111:831–6. doi: 10.1001/archopht.1993.01090060119035 8512486

[7] Shajari M Rusev V Mayer W Diakonis V Petermann K Kohnen T. Impact of lens density and lens thickness on cumulative dissipated energy in femtosecond laser-assisted cataract surgery. Lasers Med Sci. (2019) 34:1229–34. doi: 10.1007/s10103-019-02715-6, PMID: 30661184

[8] Charles Crozafon P Bouchet C Zignani M Griner R Foster SD Zou M. Comparison of real-world treatment outcomes of femtosecond laser-assisted cataract surgery and phacoemulsification cataract surgery: a retrospective, observational study from an outpatient clinic in France. Eur J Ophthalmol. (2021) 31:1809–16. doi: 10.1177/1120672120925766, PMID: 32452248

[9] Dick HB Schultz T. On the way to zero phaco. J Cataract Refract Surg. (2013) 39:1442–4. doi: 10.1016/j.jcrs.2013.07.004, PMID: 23896387

[10] Rivera RP Hoopes PC Linn SH Hoopes PC. Comparative analysis of the performance of two different platforms for femtosecond laser-assisted cataract surgery. OPTH. (2016) 10:2069–78. doi: 10.2147/OPTH.S115483, PMID: 27799734 PMC5077266

